# Effects of fatty acid metabolites on nocturia

**DOI:** 10.1038/s41598-022-07096-5

**Published:** 2022-02-23

**Authors:** Tatsuya Ihara, Hiroshi Shimura, Sachiko Tsuchiya, Mie Kanda, Satoru Kira, Norifumi Sawada, Masayuki Takeda, Takahiko Mitsui, Eiji Shigetomi, Yoichi Shinozaki, Schuichi Koizumi

**Affiliations:** 1 Department of Urology, Interdisciplinary Graduate School of Medicine, University of Yamanashi, 1110 Shimokato, Chuo, Yamanashi 409-3898 Japan; 2 Department of Neuropharmacology, Interdisciplinary Graduate School of Medicine, University of Yamanashi, Chuo, Yamanashi Japan

**Keywords:** Urology, Urogenital diseases, Metabolism, Fat metabolism

## Abstract

Dysregulation of circadian rhythm can cause nocturia. Levels of fatty acid metabolites, such as palmitoylethanolamide (PEA), 9-hydroxy-10E,12Z-octadecadienoic acid (9-HODE), and 4-hydroxy-5E,7Z,10Z,13Z,16Z,19Z-docosahexaenoic acid (4-HDoHE), are higher in the serum of patients with nocturia; however, the reason remains unknown. Here, we investigated the circadian rhythm of fatty acid metabolites and their effect on voiding in mice. WT and *Clock* mutant (*Clock*^*Δ19/Δ19*^) mice, a model for nocturia with circadian rhythm disorder, were used. Levels of serum PEA, 9-HODE, and 4-HDoHEl were measured every 8 h using LC/MS. Voiding pattern was recorded using metabolic cages after administration of PEA, 9-HODE, and 4-HDoHE to WT mice. Levels of serum PEA and 9-HODE fluctuated with circadian rhythm in WT mice, which were lower during the light phase. In contrast, circadian PEA and 9-HODE level deteriorated or retreated in *Clock*^*Δ19/Δ19*^ mice. Levels of serum PEA, 9-HODE, and 4-HDoHE were higher in *Clock*^*Δ19/Δ19*^ than in WT mice. Voiding frequency increased in PEA- and 4-HDoHE-administered mice. Bladder capacity decreased in PEA-administered mice. The changes of these bladder functions in mice were similar to those in elderly humans with nocturia. These findings highlighted the novel effect of lipids on the pathology of nocturia. These may be used for development of biomarkers and better therapies for nocturia.

## Introduction

The prevalence of nocturia, in which an individual has to wake up at night one or more times for voiding^1^, increases with aging. Nocturia not only results in various complications, such as falls and fractures, reduction in work productivity, depression, and increased mortality, but also accounts for large economic loss^2^. Although treatment of nocturia is extremely important, it is often challenging because of the multiple causes underlying the condition^3^.

Clock genes regulate circadian rhythms in organisms, including many aspects of behavior and physiological processes involving various metabolic enzymes, channels, and receptors. Hence, abnormalities in clock genes are associated with various diseases^4^. Lower urinary tract functions also follow a circadian rhythm regulated by clock genes; for example, circadian bladder capacity is related to *Connexin 43* expression rhythm in bladder smooth muscle^5^, and circadian urinary sensation is related to the rhythms of mechanosensitive ion channels in bladder urothelial cells^6–9^, which is an integral part of a sensory receptor^10^. These circadian bladder functions deteriorate with abnormalities in clock genes, and reports have shown the relationship between circadian rhythm disorders due to clock gene abnormalities and nocturia^11^. Studies have revealed that a misaligned circadian rhythm can be altered to regular circadian rhythm by drugs that act on clock genes, and that nocturia was also ameliorated with modification of the circadian rhythm^12^. These reports strengthen the relationship between nocturia and circadian rhythm.

Clock genes are also extremely important for the formation of regular lipid metabolic rhythms. Lipid metabolism is regulated by clock genes via lipid synthetic and metabolic enzymes, which lead to the circadian regulation of lipid levels^13^. In addition, a disrupted circadian clock influences circadian regulation of lipid metabolic pathways. Although the serum concentrations of various types of lipids, including fatty acids, triglycerides, glycerophospholipids, sterol lipids, and sphingolipids, have been reported to vary with circadian rhythm^13–15^, the types of lipids that are critically controlled by clock genes remain unclear because of strong differences among individuals^16^. Recently, we investigated the association between serum metabolites and nocturia in the elderly using metabolomics analysis. In this pilot study, the levels of some fatty acid metabolites, such as palmitoylethanolamide (PEA), 9-hydroxy-10E,12Z-octadecadienoic acid (9-HODE), and 4-hydroxy-5E,7Z,10Z,13Z,16Z,19Z-docosahexaenoic acid (4-HDoHE), were shown to be higher in the plasma of patients with nocturia than in that of those without nocturia^17^. Various fatty acid metabolites have been reported to regulate intracellular signaling, transcription factors, and gene expression^18^. The identified fatty acid metabolites, including PEA, 9-HODE, and 4-HDoHE, may also be involved in the development of nocturia with a background of circadian rhythm disorder. However, the physiological roles of lipids in the urinary tract are not known, because of which research on lipid-mediated regulation of lower urinary tract function is considered promising^19^. Here, we hypothesized that dysregulation of clock genes causes nocturia via elevation of fatty acid metabolite levels during sleep. Hence, we investigated the circadian changes in the levels of serum fatty acid metabolites and their effects on voiding behavior, such as nocturia, in mice. The results obtained may lead to the novel effect of lipids on lower urinary tract function and a new pathology for nocturia.

## Results

### Levels of fatty acid metabolites changed with circadian rhythm in the sera of wild-type (WT) mice and deteriorated in those of ***Clock*** mutant mice (***Clock***^***Δ19/Δ19***^)

**Figure 1:**
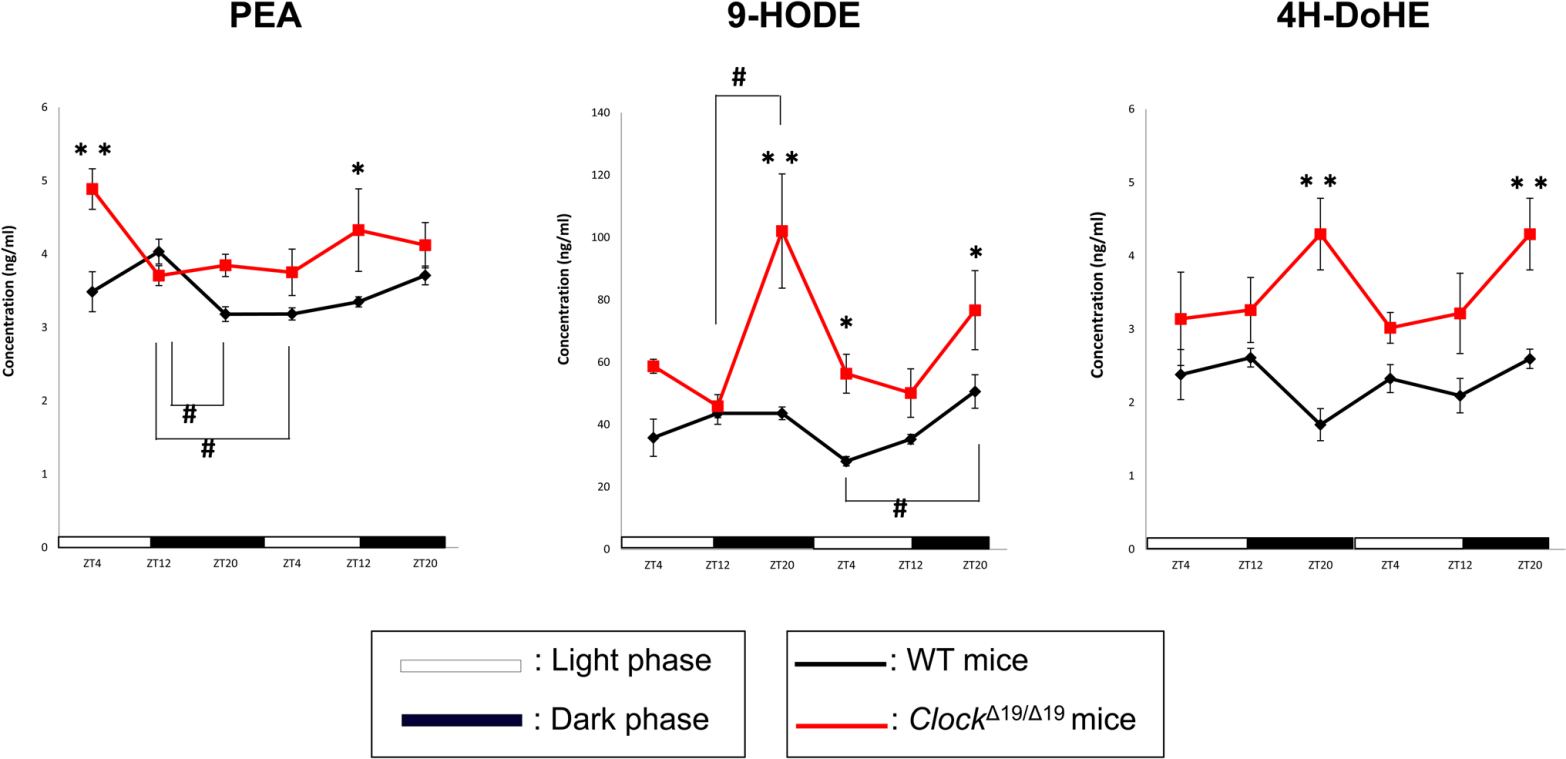
Levels of fatty acid metabolites in mouse serum. Data are presented as means ± standard error (SE). Four mice were used at each time point. One-way ANOVA with Bonferroni’s post hoc test was used to compare the differences in time-dependent change in each group. Two-way ANOVA with Bonferroni’s post hoc test was used to compare the differences at each time-point between WT and *Clock*^*Δ19/Δ19*^ mice. *p* < 0.05 was considered significant. ^#^*p* < 0.05 using one-way ANOVA with Bonferroni’s post hoc test. *p < 0.05; **p < 0.01 using two-way ANOVA with Bonferroni’s post hoc test. ZT, Zeitgeber time.

PEA, 9-HODE, and 4-HDoHE levels were measured in mouse sera using LS/MS (Fig. 1). The serum PEA level in WT mice and serum 9-HODE level in WT and *Clock*^*Δ19/Δ19*^ mice changed with circadian rhythm (*p* = 0.021, 0.027, and 0.029 for the serum PEA levels of WT mice, serum 9-HODE levels of WT mice, and serum 9-HODE levels of *Clock*^*Δ19/Δ19*^ mice using one-way ANOVA with Bonferroni’s post hoc test, respectively). However, a phase delay was observed in the circadian rhythm of the serum 9-HODE level in *Clock*^*Δ19/Δ19*^ mice compared to that in WT mice. The serum 4H-DoHE level did not fluctuate with time both in WT and *Clock*^*Δ19/Δ19*^ mice. The levels of these fatty acid metabolites were higher in *Clock*^*Δ19/Δ19*^ mice than in WT mice. The serum PEA level during the light phase [Zeitgeber time (ZT) 4 on day 1 and at the end of the light phase (ZT12 on day 2)] and serum 4-HDoHE level during the dark phase (ZT20 on day 1 and 2) were higher in *Clock*^*Δ19/Δ19*^ mice than in WT mice, whereas the serum 9-HODE level was higher in both the dark (ZT20 on day1 and 2) and light phases (ZT4) in *Clock*^*Δ19/Δ19*^ than in WT mice.

### Intraperitoneal injection of the fatty acid metabolites induced voiding reflex in WT mice under a constant dark cycle

**Figure 2:**
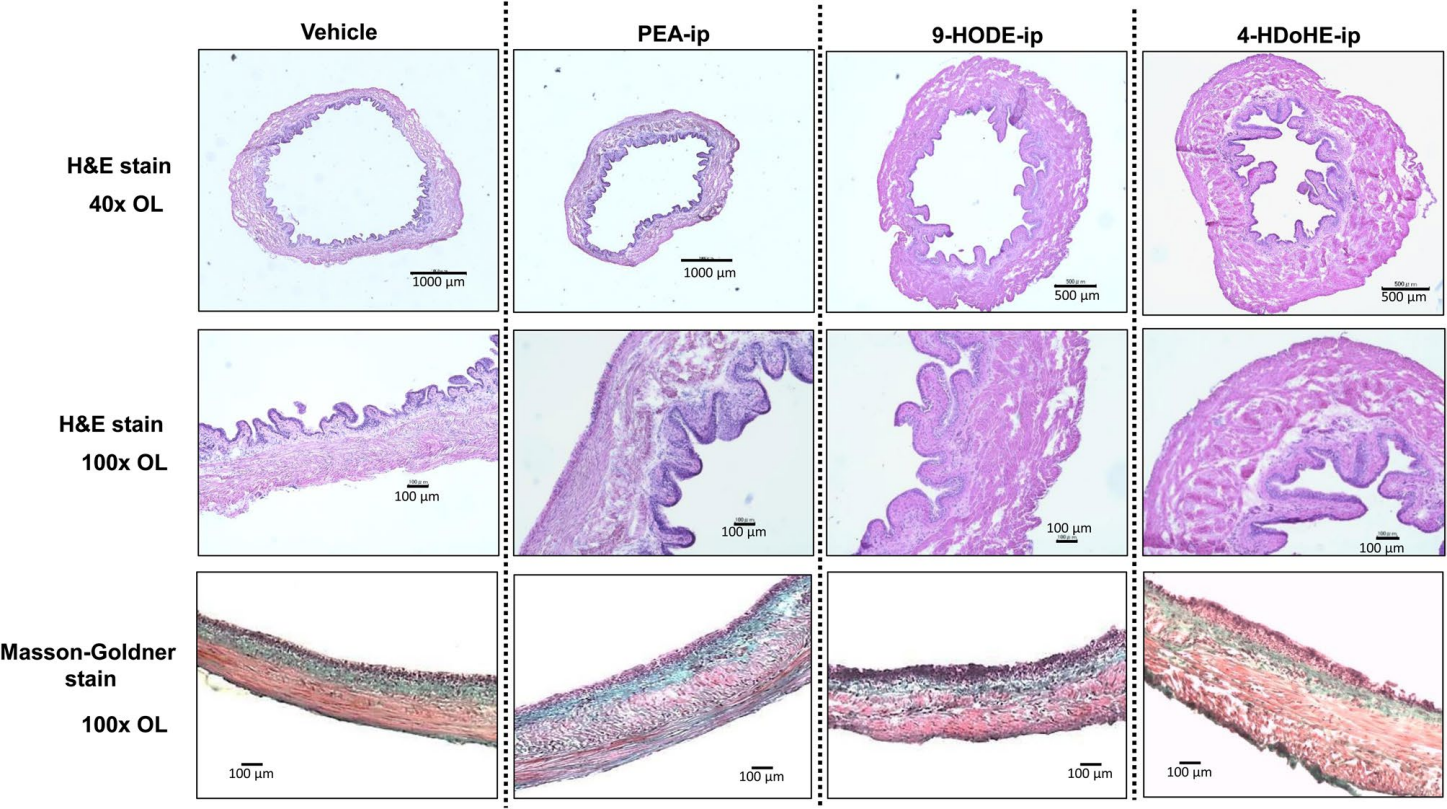
Histological examination of mouse bladder. Bladder tissue stained with hematoxylin and eosin (H & E) and Masson–Goldner stain in wild type mice injected with the vehicle and after ip of PEA (PEA-ip), 9-HODE (9-HODE-ip), and 4-HDoHE (4-HDoHE-ip). 40 × OL indicates 40 times magnification by optical lens.

As a pharmacokinetics study, 1 mg/kg each of PEA, 9-HODE, and 4-HDoHE was administered to WT mice intraperitoneally (Supplementary Information 1). A substantial increase in the serum levels of these metabolites was observed after injection. Next, 10 mg/kg each of PEA, 9-HODE, and 4-HDoHE was administered to WT mice under the 12 h light/dark cycle (LD) and constant dark cycle (DD) conditions and to *Clock*^*Δ19/Δ19*^ mice under DD condition. The number of mice used in the present study and the body weights in each group are shown in Supplementary Information 2. Mucosal exfoliation, edematous change, and inflammatory cell infiltration were not observed in the bladder tissue in the vehicle after intraperitoneal administration of PEA (PEA-ip), 9-HODE (9-HODE-ip), and 4-HDoHE (4-HDoHE-ip) (Fig. 2). In *Clock*^*Δ19/Δ19*^ mice, the voiding behavior under DD condition did not differ after the intraperitoneal administration of each fatty acid metabolite (Supplementary Information 3).

**Figure 3:**
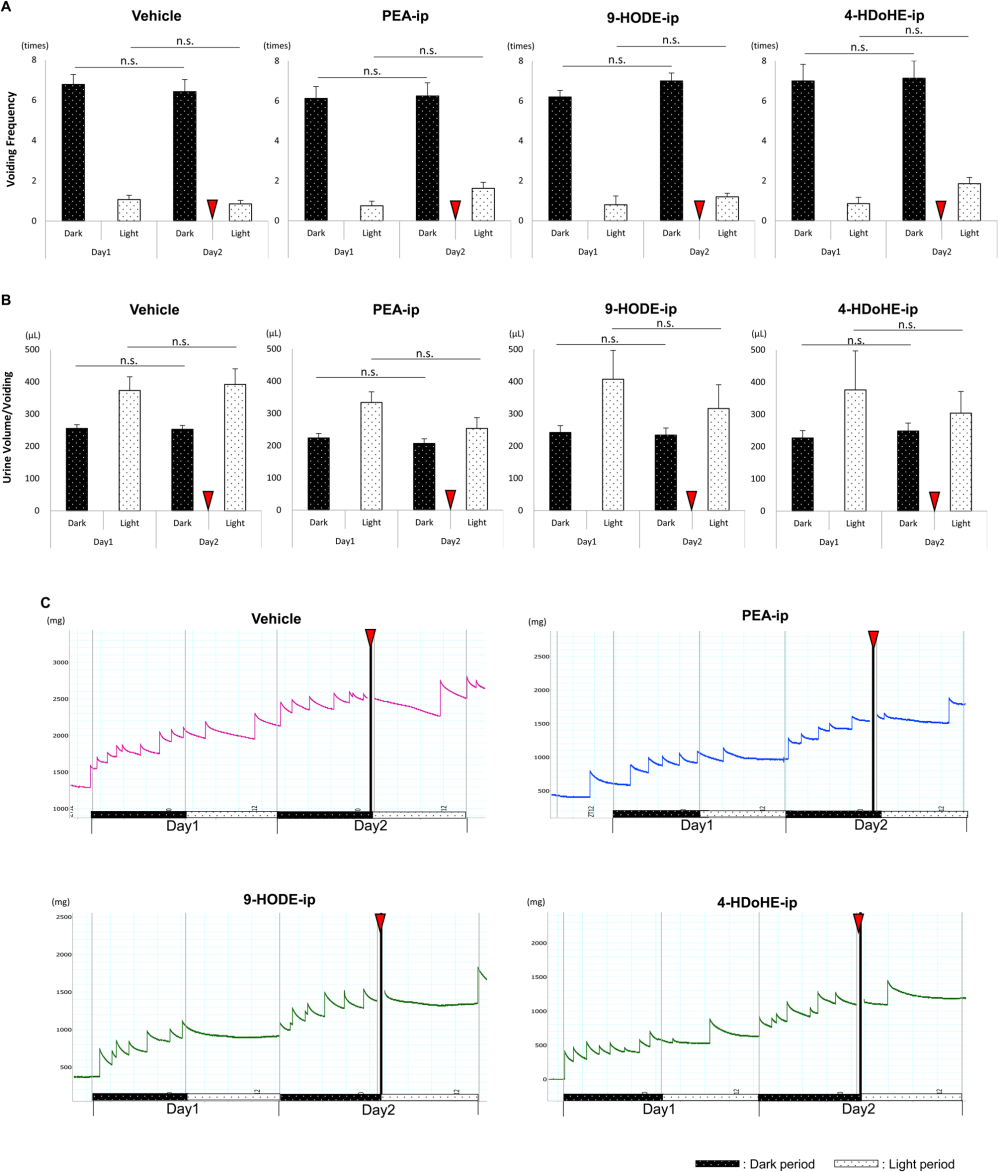
Differences in voiding behavior after intraperitoneal injection of the fatty acid metabolites under the 12 h light/dark cycle (LD) in wild type mice. Voiding frequency (VF) and urine volume per voiding (Uvol/v) were measured for 2 days and the parameters between the same phases (the dark phase on day 1 and day 2, or the light phase on day 1 and day 2) were compared. The intraperitoneal injections of vehicle, 10 mg/kg of PEA (PEA-ip), 10 mg/kg 9-HODE (9-HODE-ip), and 10 mg/kg of 4-HDoHE (4-HDoHE-ip) were performed at the beginning of the light phase on day 2. (**A**) VF between the dark and light phases for 2 days in wild type (WT) mice. (**B**) Uvol/v between the dark and light phases for 2 days in WT mice. (**C**) Representative traces of voiding of WT mice after injecting the vehicle, PEA, 9-HODE, and 4-HDoHE. Differences in VF were analyzed using paired *t* test. Differences in Uvol/v were analyzed using Mann–Whitney’s *U*-test. Data are presented as mean ± standard error (SE). *p* < 0.05 was considered significant. n.s., not significant. Red arrow heads indicate the time point of ip.

Under LD condition (Fig. 3), voiding frequency (VF) and urine volume/voiding (Uvol/v) were identical in the same phases on day 1 and day 2 in the vehicle-injected WT mice. VF and Uvol/v in the dark phase on days 1 and 2 were identical after PEA-ip, 9-HODE-ip, and 4-HDoHE-ip. VF in the light phase appeared to be higher and Uvol/v in the light phase appeared to be lower after PEA-ip, 9-HODE-ip, and 4-HDoHE-ip in WT mice; however, significant differences were not observed (*p* = 0.064, 0.270, and 0.086 for VF in the light phase on days 1 and 2 after PEA-ip, 9-HODE-ip, and 4-HDoHE-ip in WT mice using paired *t* test, respectively; *p* = 0.088, 0.368, and 0.737 for Uvol/v in the light phase on days 1 and 2 after PEA-ip, 9-HODE-ip, and 4-HDoHE-ip in WT mice using Mann–Whitney’s *U*-test, respectively).

**Figure 4:**
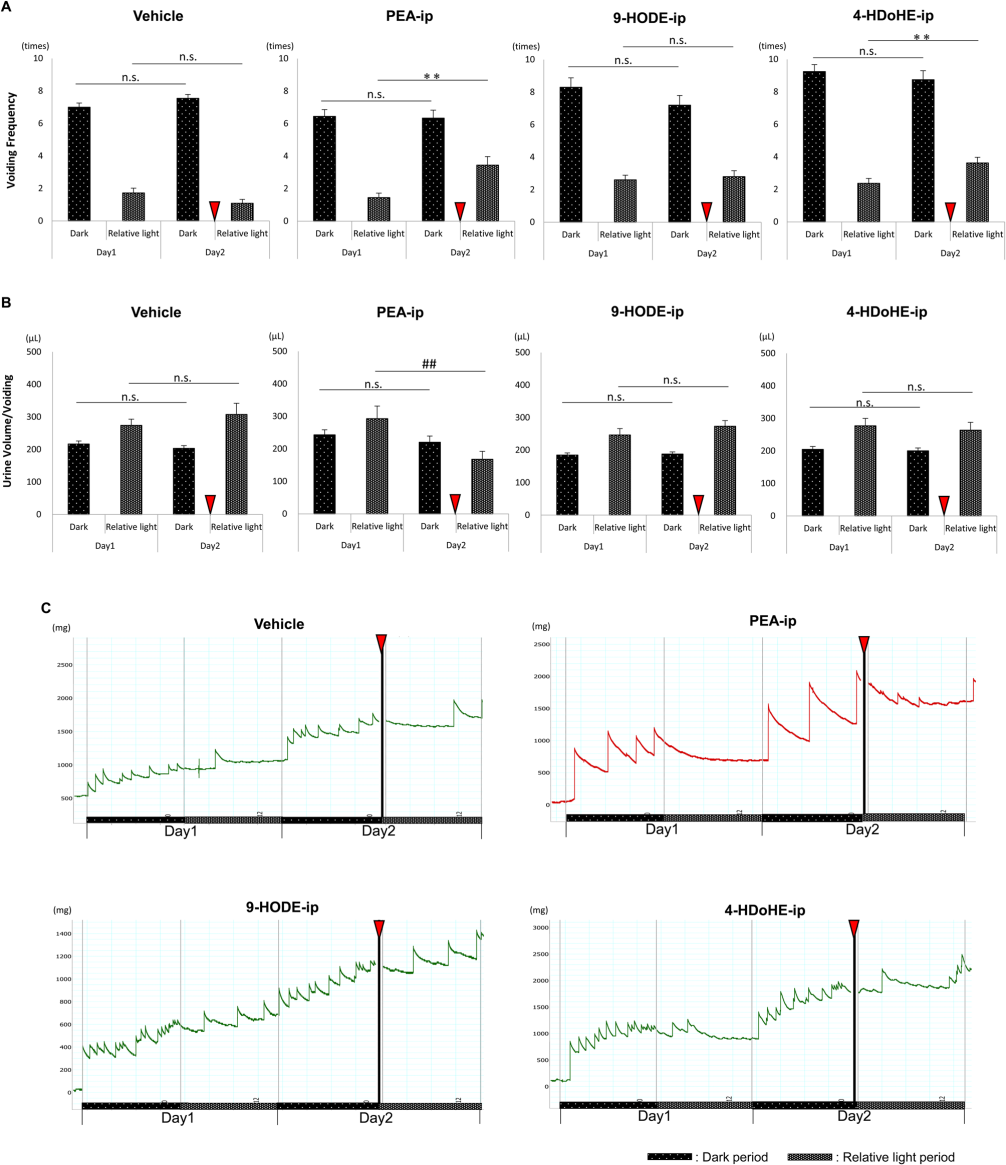
Differences in voiding behavior after intraperitoneal injection of the fatty acid metabolites under the constant dark cycle (DD) in wild type mice. Voiding frequency (VF) and urine volume per voiding (Uvol/v) were measured for 2 days and the parameters between the same phases (the dark phase on day 1 and day 2, or the relative light phase on day 1 and day 2) were compared. The intraperitoneal injections of vehicle, and 10 mg/kg each of PEA (PEA-ip), 9-HODE (9-HODE-ip), and 4-HDoHE (4-HDoHE-ip) were performed at the beginning of the relative light phase on day 2. (**A**) VF between the dark and relative light phases for 2 days in wild type (WT) mice. (**B**) Uvol/v between the dark and relative light phases for 2 days in WT mice. (**C**) Representative traces of voiding in WT mice after injecting vehicle, PEA, 9-HODE, and 4-HDoHE. Differences in VF were analyzed using paired *t* test. Differences in Uvol/v were analyzed using Mann–Whitney’s *U*-test. Data are presented as mean ± standard error (SE). *p* < 0.05 was considered significant. n.s., not significant. Red arrow heads indicate the time point of ip. **p < 0.01 by paired *t* tests, ^##^*p* < 0.01 using Mann–Whitney’s *U*-test.

Under DD condition (Fig. 4), VF and Uvol/v in the same phases on day 1 and day 2 in the vehicle-injected WT mice, and VF and Uvol/v in the dark phase on day 1 and day 2 after PEA-ip, 9-HODE-ip, and 4-HDoHE-ip in WT mice were identical, similar to that shown in Fig. 3 (Fig. 4A, B). VF in the relative light phase increased after PEA-ip and 4-HDoHE-ip (Fig. 4A) in WT mice. Uvol/v in the relative light phase decreased after PEA-ip in WT mice. However, Uvol/v in the relative light phase after 4-HDoHE-ip did not differ between day 1 and day 2 in WT mice (Fig. 3B). 9-HODE-ip in WT mice did not influence VF and Uvol/v in the relative light phase (Fig. 4A, B).

## Discussion

The present study investigated the diurnal changes in the levels of the serum fatty acid metabolites PEA, 9-HODE, and 4-HDoHE and their effects on voiding behavior. We demonstrated that the levels of PEA and 9-HODE in the serum changed with circadian rhythm in WT mice and were misaligned with the circadian rhythm in *Clock*^*Δ19/Δ19*^ mice. In addition, the serum levels of PEA, 9-HODE, and 4-HDoHE were higher in *Clock*^*Δ19/Δ19*^ mice than in WT mice. Furthermore, PEA-ip and 4-HDoHE-ip in the relative light phase triggered voiding reflex in WT mice. These results indicated that an increase in the serum level of fatty acid metabolites during sleep due to circadian rhythm disorders can cause nocturia. We suggested that the metabolic pathways of fatty acids, including those of PEA, 9-HODE, and 4-HDoHE, were involved in clock gene regulation; moreover, the circadian rhythm of fatty acid metabolism was strongly related to nocturia development.

*Clock*^*Δ19/Δ19*^ mice have been reported to show nocturia phenotype due to the loss of circadian bladder functions, which is characterized by lower VF and higher Uvol/v in the light phase than in the dark phase in WT mice^11^. Interestingly, the differences in the characteristics of voiding behaviors and circadian changes in the serum level of PEA, 9-HODE, and 4-HDoHE between WT and *Clock*^*Δ19/Δ19*^ mice were in agreement with the differences observed previously between elderly patients with nocturia and those without nocturia^17,20^. This is the first study to report the relationship between nocturia and levels of fatty acid metabolites, such as PEA, 9-HODE, and 4-HDoHE in the serum. As lipids are not genetically encoded, they are not directly regulated by clock genes. PEA and 9-HODE are synthesized via an oxidation reaction of palmitic acid and linoleic acid, an essential fatty acid. 4-HDoHE is synthesized during the metabolism of α-linolenic acid. The enzymes required for the synthesis of these fatty acids and the circadian rhythm associated with their expression are currently unknown. However, various enzymes involved in lipid metabolism, such as peroxisome proliferator-activated receptor (PPAR), peroxisome proliferator-activated receptor gamma coactivator 1-alpha (PGC-1α), and adipose triglyceride lipase (ATGL), are reported to be regulated by clock genes^13^, resulting in approximately 17% of total circadian lipid variation^16^. The circadian rhythm of the serum PEA and 9-HODE levels in WT mice and changes in their levels or their disappearance in *Clock*^*Δ19/Δ19*^ mice suggested that they are also regulated by clock genes*,* although indirectly, via the circadian fatty acid metabolic pathway.

G protein-coupled receptor 55 (GPR55) is a specific receptor for PEA^21,22^. GPR55 is distributed in the whole body, including the bladder^22,23^. However, as GPRs are regulated in an organ- and species-dependent manner, the role of GPR55 in individual organs is often unknown; hence, the effect of PEA on organs varies^21,24–26^. In the present study, we investigated only the circadian change in serum PEA level associated with clock genes (Fig. 1) and mice voiding induced by PEA administration (Fig. 4). It is necessary to investigate whether the downstream pathway after GPR55 activation by PEA may induce voiding reflex, in particular in the lower urinary tract. Arachidonic acid, essential for eicosanoid production, which can induce various stimuli in the urinary tract, is involved in 9-HODE production^27^. In addition, 9-HODE is reported as a ligand of GPR132^28^. Docosahexaenoic acid (DHA), essential for eicosanoid production and bioactive in itself, is involved in 4-HDoHE production^29^. Information regarding the effect of PEA, 9-HODE, and 4-HDoHE on the urinary tract is limited. However, changes in the level or rhythm of metabolic processes may affect fatty acid metabolism and indirectly nocturia, as well as the activation of unknown specific receptors of fatty acid metabolites, such as GPR55 and GPR132.

Some GPRs may mediate lower urinary tract dysfunction. Signaling via GPR91, a receptor for succinate, has been reported to occur in over active bladders^30^, which is defined as urinary urgency, usually accompanied by frequency and nocturia with or without urgent urinary incontinence^31^. Although GPR55 and GPR132 are orphan receptors, they may be associated with the pathology of nocturia. In particular, PEA, which considerably affects voidings in mice and is strongly associated with clock genes, appears to be the most important factor linking circadian rhythm disorder and nocturia.

The bladder capacity in mice follows the circadian rhythm, which is higher in the light phase than in the dark phase^11,12^. The circadian rhythm of Uvol/v was also observed in the present study (Figs. 3B, 4B), except for in *Clock*^*Δ19/Δ19*^ mice (Supplementary Information 3B). Therefore, we concluded that the recordings of voiding behavior did not reflect circadian bladder function in DD condition for the short period of 2 days. The LD condition, which is the pivotal circadian entrainment regulator, creates robust circadian rhythms of voiding in mice^11,32^, indicating that the setting of the light and dark environment is extremely important in studies on nocturia. Actually, intraperitoneal injection of fatty acid metabolites negligibly affected voiding behavior under LD condition (Fig. 3). This suggested the importance of LD condition in the formation of circadian voiding rhythm, especially, in the physiology of nocturia. In addition, food and drink were freely available in the present study. However, time-restricted feeding in the active phase improves circadian rhythms in metabolic regulators and clock genes^33^. More effects of fatty acid metabolites on voiding might be observed under different conditions, such as feeding restriction, which must be investigated not only after one time administration of lipids, but also more sequentially.

In addition to PEA, 9-HODE, and 4-HDoHE, the levels of various fatty acid metabolites are also elevated during nocturia. We selected these three fatty acid metabolites as targets most closely associated with nocturia, with reference to the results of metabolomics analysis^17^. Most fatty acid metabolites are believed to be produced during the process of circadian lipid metabolism; however, the physiological effects of these lipids on the urinary tract have not been determined. Interestingly, the initial experiment was performed with intraperitoneal injection of 1 mg/kg each of PEA, 9-HODE, and 4-HDoHE since a low-dose injection of these fatty acid metabolites could induce sufficient levels in serum (Supplementary Information 1). However, intraperitoneal injection of 1 mg/kg each of PEA, 9-HODE, and 4-HDoHE did not affect urination behavior in mice. Significant differences could be obtained with a dose of 10 mg/kg and DD conditions. Regarding PEA, its affinity to GPR55 is low, and high PEA concentrations in the plasma may be essential to activate GPR55^34^. Excessive concentrations of certain fatty acids may affect blood levels of other fatty acids, such that fatty acids as ligands may interact with each other due to the abnormal fatty acid blood concentration rhythm throughout the lipid metabolism process.

The pathology of nocturia is also exacerbated by metabolic syndromes, including obesity, hypertension, and diabetes. Dyslipidemia is a metabolic syndrome that contributes to nocturia development by interacting with other metabolic syndromes^35^. Palmitic acid, which is the most common fatty acid in the body and essential fatty acid and is widely used as a supplement and a source of 9-HODE and 4-HDoHE, seems to be a cause of nocturia due to hyperlipidemia. However, there are few reports showing that treatment of hyperlipidemia can improve nocturia^35^. These facts also indicate the particular receptors of PEA, 9-HODE, and 4-HDoHE as weak receptors. Hence, further studies are required to investigate the reason underlying the subtle differences observed during intraperitoneal injection of PEA, 9-HODE, and 4-HDoHE under low-dose injection and LD conditions.

The association between clock gene abnormalities and nocturia has been reported. Shift works, aging, and metabolic syndrome cause clock gene abnormalities and nocturia in both human and animal models^12,36^. We speculated that the rhythmicity of PEA and 9-HODE accumulation in the serum can be used as a biomarker for nocturia, as the importance of chronotherapy in nocturia has also been reported^36^. Many nocturia treatments are often ineffective because of their multifactorial and complex physiology. It is believed that a more effective treatment for nocturia can be achieved by finding an appropriate administration time for drugs according to the individual's circadian rhythm^36^. For measuring an individual's circadian rhythm, markers and methods that can reliably measure only the circadian rhythm of the urinary tract are indispensable, and the circadian levels of fatty acid metabolites in the serum, such as those of PEA and 9-HODE, can be used as candidate markers for this purpose.

The study has certain limitations. Although, we proposed several receptors that could mediate the effect of metabolites on the urinary tract, the detailed pharmacokinetics and pharmacodynamics of PEA, 9-HODE, and 4-HDoHE were not investigated in the present study. Thus, new investigations regarding the effects of these fatty acid metabolites on the urinary tract are required.

## Conclusions

Abnormalities in clock genes cause circadian misalignment and result in sustained elevation of fatty acid metabolites levels, such as those of PEA, 9-HODE, and 4-HDoHE, thereby inducing nocturia. These findings underline a new mechanism of nocturia and may be possibly used for development of lipid metabolite biomarkers of nocturia and better therapies for nocturia, such as therapeutic targets and chronotherapy.

## Materials and methods

### Animals

Eight- to twelve-week-old male C57BL/6 mice (WT) and age- and sex-matched C57BL/6 *Clock* mutant mice (*Clock*^*Δ19/Δ19*^), which exhibited nocturia^11^, were used in subsequent experiments. Mice were bred under 12 h light/dark conditions with free access to food and water. The light period started from 6 am ZT 0. Mice were sacrificed via cervical dislocation after anesthesia using 3% sevoflurane and 40 mg/kg pentobarbital. All procedures were conducted in accordance with the Guiding Principles for the Care and Use of Animals in the Field of the Physiologic Society of Japan and the policies of the Institutional Animal Care and Use Committee. In addition, all experimental protocols were approved by the Animal Care Committee of the University of Yamanashi (Chuo, Yamanashi, Japan). The study was carried out in compliance with the ARRIVE guidelines.

### Preparation of in vivo administered drugs

PEA, 9-HODE, and 4-HDoHE were purchased from Cayman Chemical (Ann Arbor, MN, USA). PEA was diluted with 10% dimethyl sulfoxide (DMSO), 10% cremophor EL® (Nacalai Tesque Inc., Kyoto, Japan), and 90% deionized water (DW). A solution of 9-HODE and 4-HDoHE in ethanol was dried under N_2_ with TurboVap® LV (Biotage, Uppsala, Sweden) and then diluted with 10% DMSO, 10% cremophor EL® (Nacalai Tesque), and 90% DW. Mice not receiving treatment received the vehicle (control mouse) [10% DMSO, 10% cremophor EL® (Nacalai Tesque Inc.), and 90% DW].

### Mouse serum sampling

Mice were anesthetized using sevoflurane in O_2_ and an intraperitoneal injection of pentobarbital. Approximately 1 mL mouse blood was obtained via cardiac puncture at ZT4, ZT12, and ZT20 for 2 days and then transferred to 1.5 mL micro tubes. The tubes were incubated for 30 min at 25 °C, and the supernatants obtained after centrifugation at 1500 rpm, 4 °C, for 20 min were stored at − 80 °C. Fifty microliters of the obtained serum samples were used for liquid chromatography-mass spectrometry (LC/MS) after protein precipitation and centrifugation at 9500 rpm, 4 °C, for 3 min.

### LC/MS

Chromatographic separation was performed using Triple Quad 5500 (AB Sciex, Framingham, MT) coupled to Shimadzu NEXERA T503N1 (Shimadzu Corp, Kyoto, Japan) and a Kinetex C8 column (Phenomenex, Torrance, CA, USA), which were connected via electrospray ionization at 40 °C in a thermostatic chamber using 0.1% formic acid in 25% acetonitrile (mobile phase A) and 0.1% formic acid in acetonitrile (mobile phase B) at a flow rate of 0.4 mL/min. The samples for LS/MS included 50 μL each of mouse serum, standard solution, and internal standard solution consisting of 10 ng/mL PEA-d4 (Cayman Chemical) and 200 ng/mL 9-HODE-d4 (Cayman Chemical); 200 μL methanol was mixed with 900 μL tert-butyl methyl ether, vortexed, and centrifuged at 18,700 rpm, 4 °C, for 5 min. An 800 μL aliquot of the supernatant was transferred to a polypropylene tube and dried under N_2_ with TurboVap® LV (Biotage). Then, 100 μL of 25% acetonitrile was added. Five microliters of the prepared samples, stored at 15 °C, were injected into the columns during the 22.5th min of gradient time. The peaks were extracted to obtain peak information, including *m/z*, LC retention time, and peak area. Quantification was performed using a calibration curve of concentration ratio vs. peak area ratio. Calibration standards with the following concentrations were prepared: PEA: 100, 30, 10, 3, 1, 0.3 ng/mL; 9-HODE: 1000, 300, 100, 30, 10, 3 ng/mL; 4-HDoHE: 100, 30, 10, 3, 1, 0.3 ng/mL. The Analyst Ver. 1.6.2 software was used for data collection and processing (AB Sciex).

### Metabolic cages

**Figure 5:**
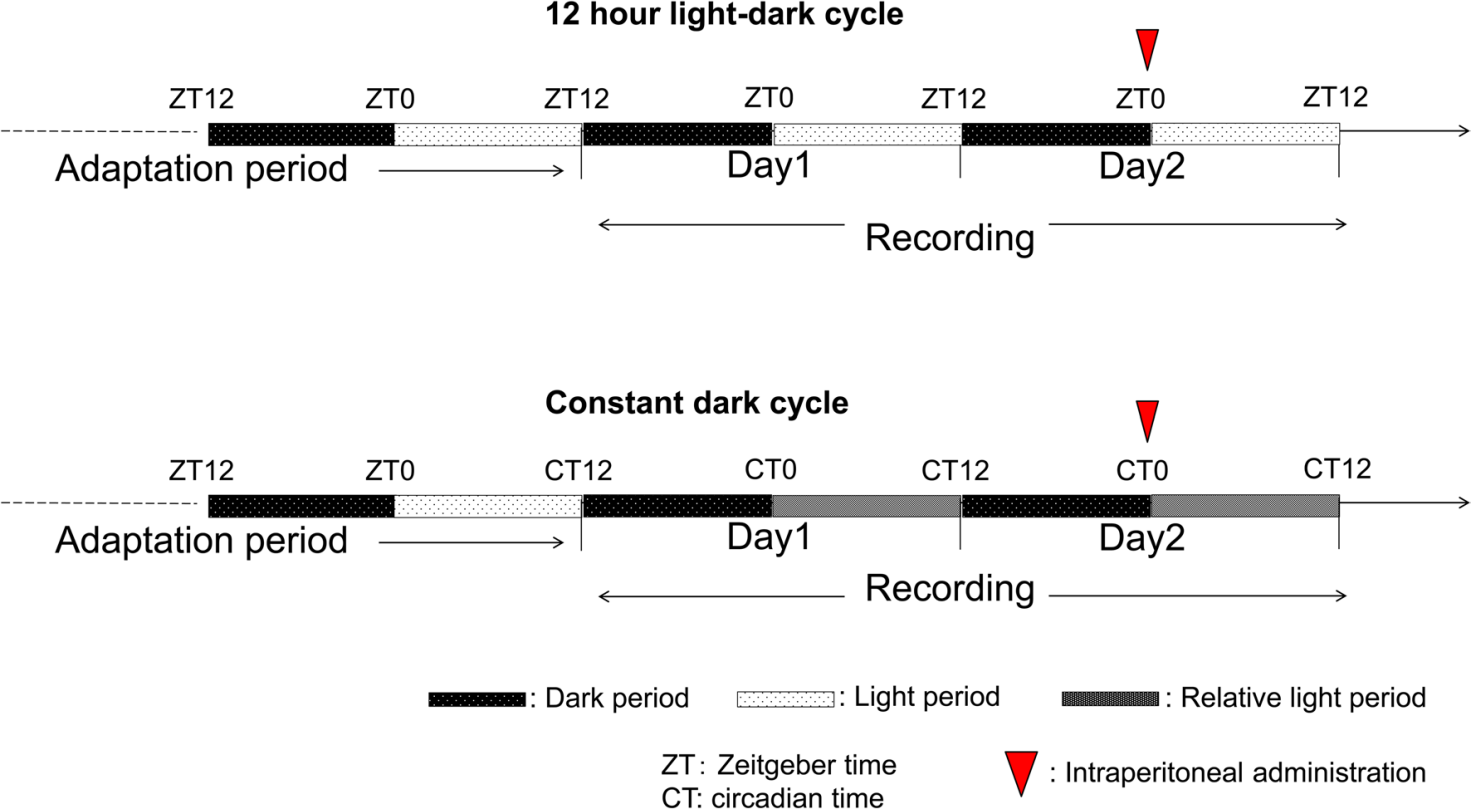
Time-course of intraperitoneal injection and recording of voiding behavior.

The voiding behaviors were recorded using metabolic cages (Shinfactory Co. Ltd., Fukuoka, Japan)^37^. The following parameters were evaluated: Uvol/v (µL) and VF (times). After the mice were acclimatized for 2 days in the cage, voiding behavior was recorded for two days under the LD and DD conditions. In total, 10 mg/kg each of PEA, 9-HODE, and 4-HDoHE was administered intraperitoneally at ZT0 under LD condition and circadian time (CT) 0 under DD condition on day 2 (Fig. 5). Voiding during the light phase under LD condition and 12 h after CT0 under DD condition (the relative light phase) was considered nocturia in mice, as defined previously^11^.

### Histological examination of mouse bladder

Mouse bladders were harvested after the recordings of voiding behavior under anesthesia. Frozen tissues embedded in OCT compound (Sakura Finetek Japan, Tokyo, Japan) were cut into 7 µm thick sections. Standard hematoxylin and eosin (H & E) and Masson–Goldner staining was performed to compare morphological changes after intraperitoneal administration of the fatty acid metabolites.

### Statistical analyses

The experimental values are expressed as means ± standard error (SE). The significance of the differences between the two groups were analyzed using paired *t* test and Mann–Whitney’s *U*-test depending on the distribution of the samples. One-way analysis of variance (ANOVA) with Bonferroni’s post hoc test was used to compare differences in time-dependent change in each group. Two-way ANOVA with Bonferroni’s post hoc test was used to compare differences among the time points between groups. Differences with P value less than 0.05 were considered significant.

## Supplementary Information

Supplementary Information.

## Abbreviations

*Clock*^*Δ19/Δ19*^: *Clock* mutant
CT: Circadian time
DD: Constant dark cycle
DMSO: Dimethyl sulfoxide
DW: Deionized water
GPR: G protein-coupled receptor
H&E: Hematoxylin and eosin
ip: Intraperitoneal injection
LC/MS: Liquid chromatography–mass spectrometry
LD: 12-Hour light/dark cycle
PEA: Palmitoylethanolamide
SE: Standard error
Uvol/v: Urine volume/voiding
VF: Voiding frequency
WT: Wild type
ZT: Zeitgeber time
4-HDoHE: 4-Hydroxy-5E,7Z,10Z,13Z,16Z,19Z-docosahexaenoic acid
9-HODE: 9-Hydroxy-10E,12Z-octadecadienoic acid

## References

[1] van Kerrebroeck P (2002). The standardisation of terminology in nocturia: Report from the Standardisation Sub-committee of the International Continence Society. Neurourol. Urodyn..

[2] Oelke M (2017). A practical approach to the management of nocturia. Int. J. Clin. Pract..

[3] Oelke M, Adler E, Marschall-Kehrel D, Herrmann TR, Berges R (2014). Nocturia: State of the art and critical analysis of current assessment and treatment strategies. World J. Urol..

[4] Okamura H, Doi M, Fustin JM, Yamaguchi Y, Matsuo M (2010). Mammalian circadian clock system: Molecular mechanisms for pharmaceutical and medical sciences. Adv. Drug Deliv. Rev..

[5] Negoro H (2012). Involvement of urinary bladder Connexin43 and the circadian clock in coordination of diurnal micturition rhythm. Nat. Commun..

[6] Ihara T (2017). The circadian expression of Piezo1, TRPV4, Connexin26, and VNUT, associated with the expression levels of the clock genes in mouse primary cultured urothelial cells. Neurourol. Urodyn..

[7] Ihara T (2017). Clock genes regulate the circadian expression of Piezo1, TRPV4, Connexin26, and VNUT in an ex vivo mouse bladder mucosa. PLoS ONE.

[8] Ihara T (2018). The oscillation of intracellular Ca(2+) influx associated with the circadian expression of Piezo1 and TRPV4 in the bladder urothelium. Sci. Rep..

[9] Ihara T (2018). The time-dependent variation of ATP release in mouse primary-cultured urothelial cells is regulated by the clock gene. Neurourol. Urodyn..

[10] Birder L, Andersson KE (2013). Urothelial signaling. Physiol. Rev..

[11] Ihara T (2017). The Clock mutant mouse is a novel experimental model for nocturia and nocturnal polyuria. Neurourol. Urodyn..

[12] Ihara T (2019). Intermittent restraint stress induces circadian misalignment in the mouse bladder, leading to nocturia. Sci. Rep..

[13] Gooley JJ (2016). Circadian regulation of lipid metabolism. Proc. Nutr. Soc..

[14] Gooley JJ, Chua EC (2014). Diurnal regulation of lipid metabolism and applications of circadian lipidomics. J. Genet. Genomics.

[15] Adamovich Y, Aviram R, Asher G (1851). The emerging roles of lipids in circadian control. Biochem. Biophys. Acta.

[16] Chua EC (2013). Extensive diversity in circadian regulation of plasma lipids and evidence for different circadian metabolic phenotypes in humans. Proc. Natl. Acad. Sci. USA.

[17] Kira S (2019). Liquid chromatography-mass spectrometry identification of serum biomarkers for nocturia in aged men. World J. Urol..

[18] Calder PC (2015). Functional roles of fatty acids and their effects on human health. J. Parenter. Enter. Nutr.: JPEN.

[19] O'Donnell VB, Ekroos K, Liebisch G, Wakelam M (2019). Lipidomics: Current state of the art in a fast moving field. Wiley Interdiscip. Rev. Syst. Biol. Med..

[20] Kira S (2018). Lack of change in the adaptation ability of the bladder for the urine production rate in aged men with nocturia. Urol. Int..

[21] Tuduri E (2017). GPR55: A new promising target for metabolism?. J. Mol. Endocrinol..

[22] Ryberg E (2007). The orphan receptor GPR55 is a novel cannabinoid receptor. Br. J. Pharmacol..

[23] Henstridge CM (2011). Minireview: Recent developments in the physiology and pathology of the lysophosphatidylinositol-sensitive receptor GPR55. Mol. Endocrinol. (Baltimore, Md.).

[24] Henstridge CM (2009). The GPR55 ligand L-alpha-lysophosphatidylinositol promotes RhoA-dependent Ca2+ signaling and NFAT activation. FASEB J..

[25] Leo LM (2019). GPR55-mediated effects on brain microvascular endothelial cells and the blood–brain barrier. Neuroscience.

[26] Lauckner JE (2008). GPR55 is a cannabinoid receptor that increases intracellular calcium and inhibits M current. Proc. Natl. Acad. Sci. USA.

[27] Andersson KE, Fry C, Panicker J, Rademakers K (2018). Which molecular targets do we need to focus on to improve lower urinary tract dysfunction? ICI-RS 2017. Neurourol. Urodyn..

[28] Vangaveti V (2018). 9- and 13-HODE regulate fatty acid binding protein-4 in human macrophages, but does not involve HODE/GPR132 axis in PPAR-γ regulation of FABP4. Ther. Adv. Endocrinol. Metab..

[29] Mallick R, Basak S, Duttaroy AK (2019). Docosahexaenoic acid,22:6n–3: Its roles in the structure and function of the brain. Int. J. Dev. Neurosci..

[30] Mossa AH, Velasquez Flores M, Cammisotto PG, Campeau L (2017). Succinate, increased in metabolic syndrome, activates GPR91 receptor signaling in urothelial cells. Cell. Signal..

[31] Haylen BT (2010). An International Urogynecological Association (IUGA)/International Continence Society (ICS) joint report on the terminology for female pelvic floor dysfunction. Int. Urogynecol. J..

[32] Baron KG, Reid KJ (2014). Circadian misalignment and health. Int. Rev. Psychiatry (Abingdon, England).

[33] Hatori M (2012). Time-restricted feeding without reducing caloric intake prevents metabolic diseases in mice fed a high-fat diet. Cell Metab..

[34] Tudurí E (2017). GPR55: A new promising target for metabolism?. J. Mol. Endocrinol..

[35] Aoki Y, Yokoyama O (2012). Metabolic Syndrome and Nocturia. Lower Urin. Tract symptoms.

[36] Ihara T (2021). Different effects of GsMTx4 on nocturia associated with the circadian clock and Piezo1 expression in mice. Life Sci..

[37] Yoshiyama M (2015). Functional roles of TRPV1 and TRPV4 in control of lower urinary tract activity: Dual analysis of behavior and reflex during the micturition cycle. Am. J. Physiol. Renal Physiol..

