# Supplementary material for: Effects of fatty acid metabolites on nocturia
Source: Sci Rep. 2022 Feb 23;12:3050. doi: 10.1038/s41598-022-07096-5 (PMC8866436; doi:10.1038/s41598-022-07096-5)
Supplement: Supplementary file 1 — Supplementary Information. [file 41598_2022_7096_MOESM1_ESM.doc]

**Effects of fatty acid metabolites on nocturia**

Tatsuya Ihara^1*^, Hiroshi Shimura^1^, Sachiko Tsuchiya^1^, Mie Kanda^1^, Satoru Kira^1^, Norifumi Sawada^1^, Masayuki Takeda^1^, Eiji Shigetomi^2^, Yoichi Shinozaki^2^, Schuichi Koizumi^2^, Takahiko Mitsui^1^

^1^Department of Urology, Interdisciplinary Graduate School of Medicine, University of Yamanashi, Chuo, Yamanashi, Japan

^2^Department of Neuropharmacology, Interdisciplinary Graduate School of Medicine, University of Yamanashi, Chuo, Yamanashi, Japan

*Corresponding author:

Tatsuya Ihara

1110 Shimokato, Chuo, Yamanashi, 409-3898 Japan

Tel: +81-55-273-9643

Fax: +81-55-273-9659

E-mail: [tihara@yamanashi.ac.jp](mailto:tihara@yamanashi.ac.jp)

**Supplementary Information 1. Concentrations of fatty acid metabolites after** **1 mg/kg intraperitoneal administration of PEA, 9-HODE, and 4-HDoHE in mice.**

**
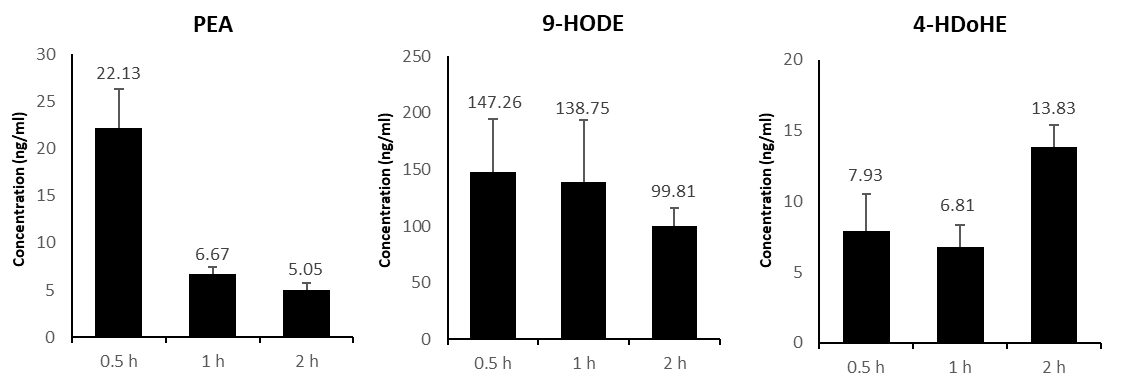
**

After the intraperitoneal injections of 1 mg/kg each of PEA, 9-HODE, and 4-HDoHE were performed for mice, mouse sera were sampled at 0.5, 1, 2 h later, and then the concentrations of each fatty acid metabolite were measured using liquid chromatography-mass spectrometry. Data are presented as means ± standard error (SE). Three mice were used at each time point.

**Supplementary Information 2. Number of mice and body weights used in the**

**present study.**


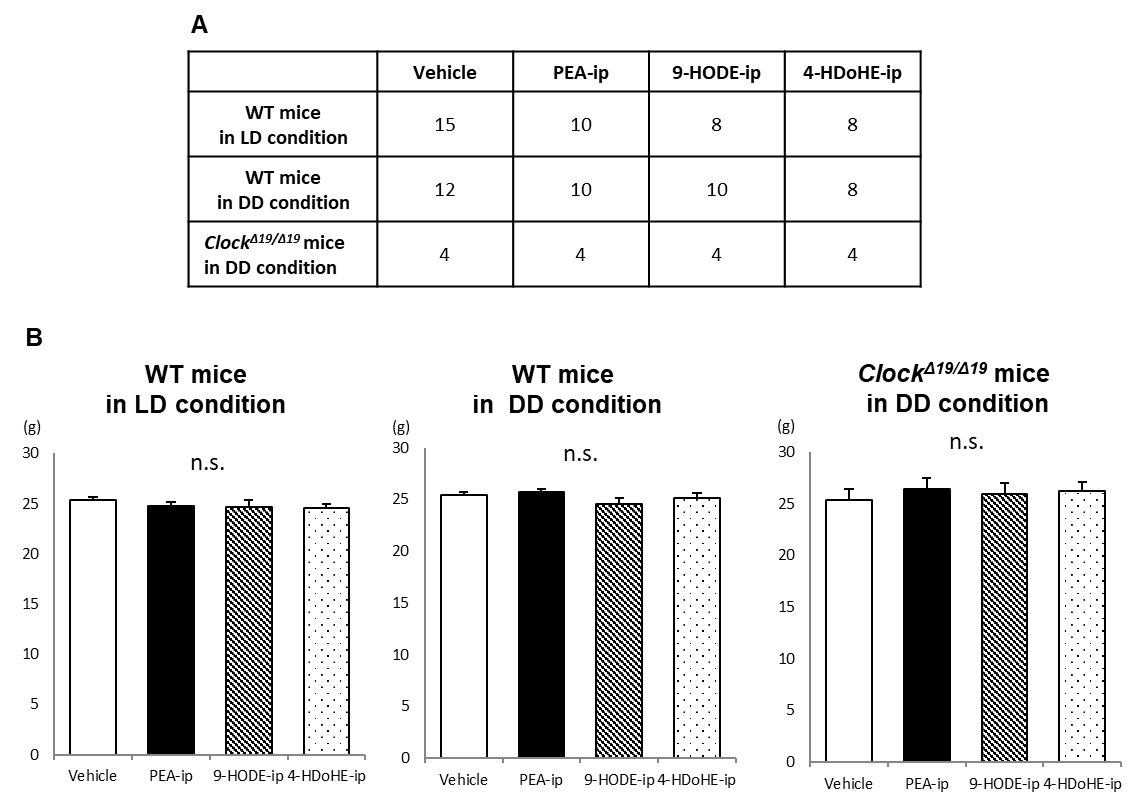


**A.** Number of mice used in the present study in each group and condition. **B.** The mean body weights for each group. The body weights were compared using one-way analysis of variance with Bonferroni’s post hoc test; differences were not observed. WT, wild-type mice; *Clock^Δ19/Δ19^*, Clock mutant mice; LD, 12-hour light-dark cycle; DD, constant dark cycle; ip, intraperitoneal injection.

**Supplementary Information 3. Differences in voiding behavior after intraperitoneal injection of the fatty acid metabolites under the constant dark cycle (DD) in *Clock^Δ19/Δ19^* mice.**


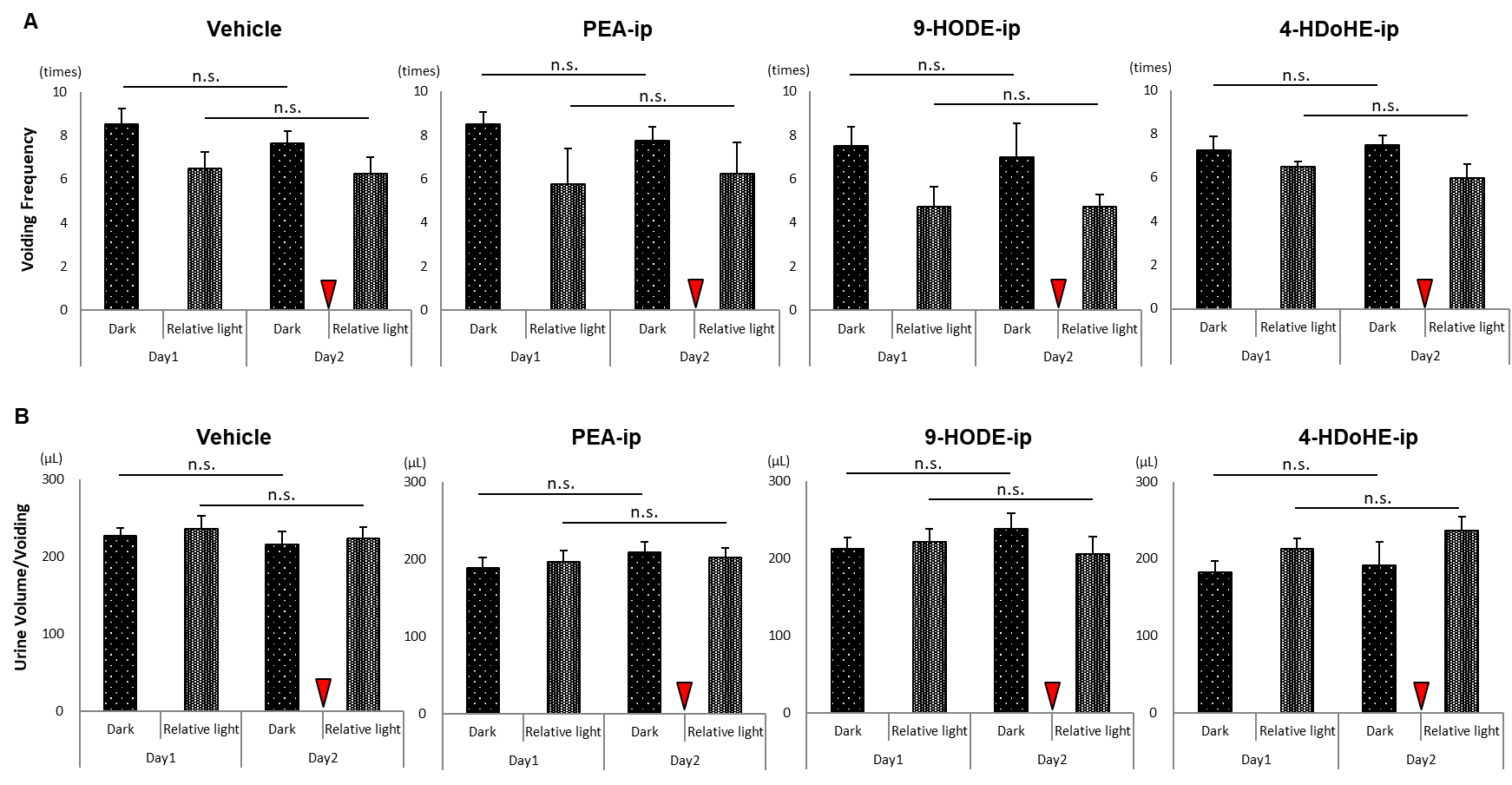


**
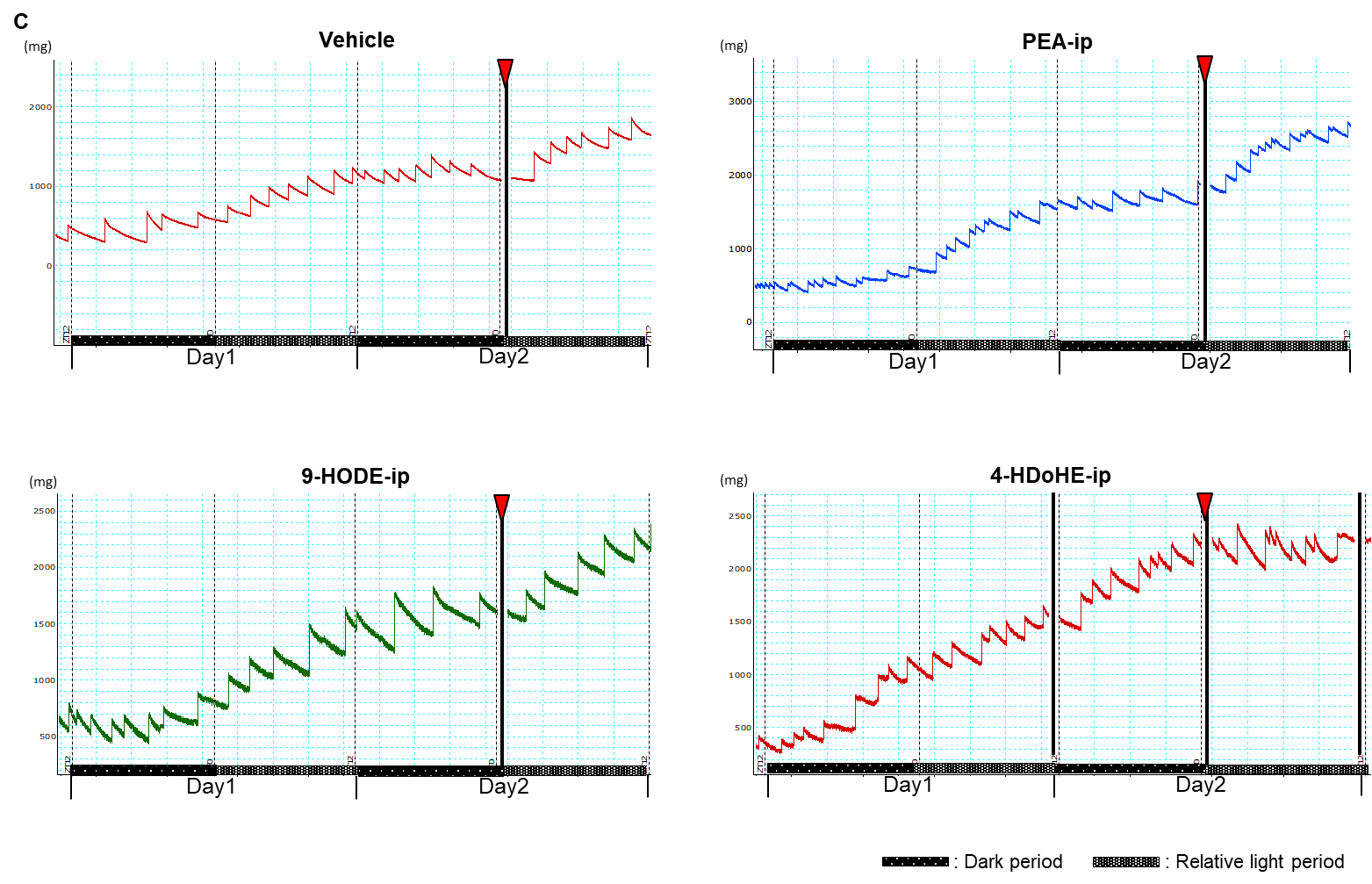
**

Voiding frequency (VF) and urine volume per voiding (Uvol/v) were measured for 2 days and the parameters between the same phases (the dark phase on day 1 and day 2, or the relative light phase on day 1 and day 2) were compared. The intraperitoneal injections of vehicle, 10 mg/kg each of PEA (PEA-ip), 9-HODE (9-HODE-ip), and 4-HDoHE (4-HDoHE-ip) were performed at the beginning of the relative light phase on day 2. **A.** VF between the dark and relative light phases for 2 days in *Clock^Δ19/Δ19^* mice. **B.** Uvol/v between the dark and relative light phases for 2 days in *Clock^Δ19/Δ19^* mice. **C.** Representative traces of voiding in WT mice injected with vehicle, PEA, 9-HODE, and 4-HDoHE. Differences in VF were analyzed using paired t-test. Differences in Uvol/v were analyzed using Mann-Whitney’s *U*-test. Data are presented as mean ± standard error (SE). p < 0.05 was considered significant. n.s., not significant; *Clock^Δ19/Δ19^*, Clock mutant. Red arrow heads indicate the time point of ip.
